# Measuring the burden of treatment for chronic disease: implications of a scoping review of the literature

**DOI:** 10.1186/s12874-017-0411-8

**Published:** 2017-09-12

**Authors:** Adem Sav, Asiyeh Salehi, Frances S. Mair, Sara S. McMillan

**Affiliations:** 1 School of Allied Health, Australian Catholic University, Banyo, Queensland Australia; 2 Menzies Health Institute Queensland, Griffith University, University Drive, Meadowbrook, QLD Australia; 3 General Practice and Primary Care, Institute of Health and Wellbeing, College of Medical, Veterinary and Life Sciences, University of Glasgow, Glasgow, Scotland, UK; 4 PO Box 456, Virginia, 4014 Australia

**Keywords:** Burden of treatment, Burden of medication, Treatment experience, Time burden, Workload burden, Cost of illness, Chronic disease

## Abstract

**Background:**

Although there has been growing research on the burden of treatment, the current state of evidence on measuring this concept is unknown. This scoping review aimed to provide an overview of the current state of knowledge as well as clear recommendations for future research, within the context of chronic disease.

**Methods:**

Four health-based databases, Scopus, CINAHL, Medline, and PsychInfo, were comprehensively searched for peer-reviewed articles published between the periods of 2000–2016. Titles and abstracts were independently read by two authors. All discrepancies between the authors were resolved by a third author. Data was extracted using a standardized proforma and a comparison analysis was used in order to explore the key treatment burden measures and categorize them into three groups.

**Results:**

Database searching identified 1458 potential papers. After removal of duplications, and irrelevant articles by title, 1102 abstracts remained. An additional 22 papers were added via snowball searching. In the end, 101 full papers were included in the review. A large number of the studies involved quantitative measures and conceptualizations of treatment burden (*n* = 64; 63.4%), and were conducted in North America (*n* = 49; 48.5%). There was significant variation in how the treatment burden experienced by those with chronic disease was operationalized and measured.

**Conclusion:**

Despite significant work, there is still much ground to cover to comprehensively measure treatment burden for chronic disease. Greater qualitative focus, more research with cultural and minority populations, a larger emphasis on longitudinal studies and the consideration of the potential effects of “identity” on treatment burden, should be considered.

## Background

Globally, chronic diseases, such as diabetes, cancer and asthma, are now in epidemic proportions [1]. With their increasing prevalence, the focus for health professionals has shifted from treating acute illness to helping their patients manage the ‘work’ of living with such conditions [2, 3]. The job of health professionals has been complicated by the fact that chronic disease rarely occurs in isolation, with many people experiencing two or more diseases concurrently, something known as multimorbidity [4]. For patients, there is not only the complexity of dealing with one chronic condition, but the work of trying to live ‘normal’ lives in the face of multimorbidity, which can be overwhelming. This further adds to the ‘work’ that patients must do to manage and live with such health conditions and the psychological distress they experience as a result.

Lately, the work that patients need to do to manage and treat chronic disease has been referred to as treatment burden [5]. Treatment burden represents the active work patients need to do including, learning about treatments and their consequences, completing administrative tasks, such as paper work, adhering to complex treatment regimens, managing medications, changing lifestyle behaviours, visiting multiple health professionals, and undertaking medical and other laboratory tests, etc. [6–8]. Treatment burden is concerned with the negative experiences resulting from the process of undertaking treatment [8]. The burden of treatment can be dependent on the type of treatment a person is undergoing. For example, chemotherapy to destroy cancer cells or dialysis treatment for kidney disease is much more invasive and burdensome compared to medication used to manage high blood pressure.

**Fig. 1:**
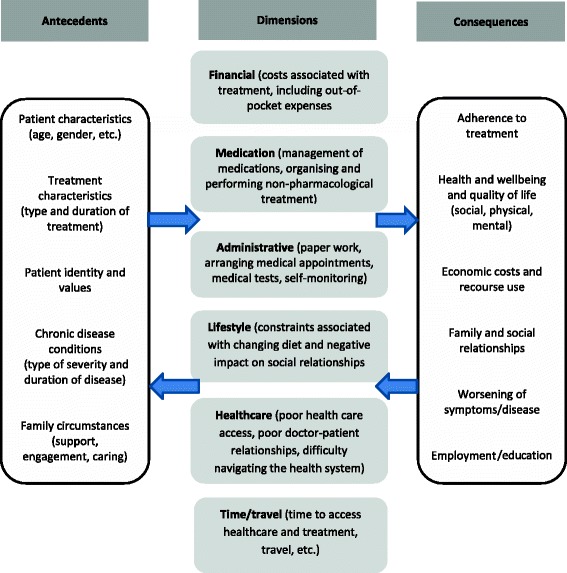
Conceptual framework for the study

Sav et al. [8] reviewed research on treatment burden published between 2000 and 2011 and suggested that a number of factors can contribute to treatment burden for chronic disease, including age and gender, illness duration or severity, treatment characteristics including the number and dose of medications, and family circumstances such as level of support. Sav et al. [8] further drew attention to the dynamic nature of treatment burden suggesting that a person’s overall perception of burden can change throughout the course of their illness, depending on its severity and impact. Tran et al. [9] in their existing taxonomy of treatment burden, indicated that healthcare which imposes a burden on patient’s include: management of medications, organising and performing non-pharmacological treatment, lifestyle changes, condition and treatment follow-up, organising formal caregiver care, paperwork tasks, and earning and developing an understanding of the illness and treatment [6]. More recently, Demain et al. [10] discussed the sociological aspect of treatment burden, suggesting that burden is not only brought about by the workload associated with a treatment regimen, but also the impact of that workload on everyday activities and a patient’s identity. For example, elderly adults may be more likely, compared to young adults, to accept treatment burden as a necessary evil brought on by old age and living with multimorbidity. Finally, treatment burden is different from other related terms, such as symptom burden, where the latter is focused on living with the burden that the disease imposes, e.g. burden on paid employment, and not on the need to treat the disease in order to change its course or ameliorate its’ effects, which is the focus of treatment burden [8]. Demain et al’s [10] Tran et al’ [9] Sav et al’s [8, 11] research in the area of treatment burden will be used as the conceptual framework to guide data collection and analysis in this manuscript (see Fig. 1). The framework, shows several antecedents, which lead to burden, the major dimensions and consequences, operating in a cyclic nature (e.g., some consequences become antecedents and vice versa). Of particular interest, the conceptual framework shows the five key dimensions of treatment burden, including, financial, medication, administrative, lifestyle, healthcare and time/travel.

Although treatment burden has gained popular research momentum in the past decade, particularly since May et al.’s seminal work in 2009 on “minimally disruptive medicine”, debate continues around the best way to measure and understand the level of treatment burden among patients. The absence of a universally accepted holistic measure makes it difficult for researchers and health professionals to understand levels of treatment burden among patients, and hinders efforts to introduce effective interventions that reduce the level of burden experienced by patients. Sav et al.’s [7] review found that treatment burden has generally been measured as one dimension, e.g. medication use, within a multidimensional instrument designed to assess health-related quality of life or treatment satisfaction. Sav et al. [8] argued that these measures had wide variation in terms of the dimensions of treatment burden and the way this concept was defined. Although significant work has been conducted on treatment burden since 2011, the current state of evidence on measuring treatment burden is unknown. Yet, such knowledge is essential in order to move forward and agree on the best way to measure treatment burden in different populations and with various or multiple chronic conditions.

In this paper we report results of a scoping review of the literature on measuring treatment burden for chronic disease. Our overarching objective is to provide an overview of the current state of knowledge on how treatment burden for chronic disease has been measured and conceptualized. It is worth noting that our aim is not to conduct an assessment of the quality of the selected literature (which is the aim of a systematic review), nor to critique the statistical assessment of treatment burden questionnaires. Rather, there was a need to ‘scope’ the large but fragmented body of literature on how a patient’s level of treatment burden, for a variety of chronic conditions, has been measured. Our secondary aims are: (i) to advance our conceptualization of the measurement of treatment burden; (ii) understand the methodological issues in developing treatment burden measurements; (iii) offer clear recommendations for future research in this sphere to develop our understanding of this concept.

## Methods

**Fig. 2:**
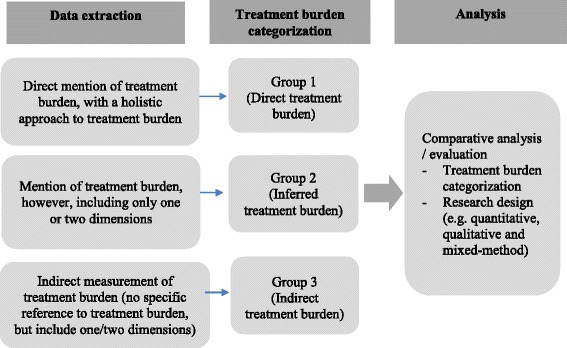
Framework of study categorization

A scoping review is a "*form of knowledge synthesis that addresses an exploratory research question aimed at mapping key concepts, types of evidence, and gaps in research related to a defined area or field by systematically searching, selecting and synthesizing existing knowledge*" [12], p. 1284. Scoping reviews can be helpful in exploring research gaps, setting research agendas and providing recommendations for policy makers [13]. Although the quality of papers are not assessed, scoping reviews explore a diverse range of papers and provide a considerable summary of the relevant literature [14–16]. Our scoping review was guided by five of the six steps outlined by Arksey and O’Malley [14]. As a widely cited and accepted way of conducting a scoping review, this framework provided an opportunity to guide the data collection and analysis in this study. The main stages included:

(i) *Classifying the research question*. The key research questions of this scoping review included (a) how was treatment burden measured? (b) what is the best way to measure this construct?
(ii) *Finding the relevant studies/search strategy*. The authors identified the main concepts of the topic, the burden of treatment in chronic health conditions, and collated a list of relevant keywords. The search strategy was refined by an initial search of the literature to identify key MESH terms relating to this topic. Four health-based databases, Scopus, CINAHL, Medline and PsychInfo, were searched using Boolean Operators for the following terms: “treatment burden” OR “burden of treatment” OR “medication burden” OR “burden of medication” OR “treatment experience” OR “time burden” OR “workload burden” OR “cost of illness” AND “chronic disease”. Snowball searching, including pursuing references of references and citations searching, was also used.
(iii) *Choosing the studies based on inclusion/exclusion criteria*. Inclusion and exclusion criteria were developed by the authors during several meetings and are described in Fig. 2 and guided by the conceptual framework developed for this study (Fig. 1). For example, to be included in the review, papers needed to measure or focus on specific dimensions of treatment burden, developed in the conceptual framework (e.g. financial, medication, administrative, lifestyle, healthcare and time/travel). Peer-reviewed journal papers were included if they were: published between the period of 2000–2016, written in English, involved human participants and described a measure for burden of treatment, e.g. including single measurements, measuring and/or incorporating one or two dimensions of burden of treatment. Quantitative, qualitative and mixed-method studies were included in order to consider different aspects of measuring treatment burden. Papers were excluded if they did not fit into the conceptual framework of the study, focused on a communicable chronic condition, for example human immunodeficiency virus infection and acquired immune deficiency syndrome (HIV/AIDS) or substance abuse. Papers talking about carer burden, in addition to patient burden of treatment, were also included.
(iv) Titles and abstracts were independently read by two authors (AS & SM) using the inclusion criteria. After applying the eligibility criteria, all remaining papers were evenly allocated between three authors (AS, NS and SM) and categorized into included, excluded or unsure. Full-text of all unsure papers was read by another author (AS, NS or SM). In case of any disagreement, the matter was resolved through in-depth discussion between the authors. *Charting the data.* Data was extracted using a standardized proforma, which collected data on: year of publication, authors, country, data collection, participant number and chronic disease. The papers were divided between three authors and the extracted data from all the papers (particularly the treatment burden categorization), were cross-checked in order to reduce any possible individual bias [17]. Disagreements regarding the treatment burden categories and data extractions were resolved by discussions and brainstorming among two authors, and then by involving a third author if required [17].
(v) *Collating, summarizing, and reporting the findings*. For all included papers, the instruments, and the key dimensions of treatment burden, or both, were described and compared. A comparison analysis was used in order to explore the key treatment burden measures and categorize the papers into three groups (with the aid of the conceptual framework), depending on the level of treatment burden measured. The three groups were:

- Direct measures (Group 1): The paper clearly states that it measures and/or explores treatment burden. This category includes a more holistic approach to treatment burden and measures all or most of the dimensions in the conceptual framework developed for this study
- Inferred measures (Group 2): The paper stated that it measured treatment burden, however, it included only one or two dimensions (which are included in the conceptual framework), such as financial burden, burden of time/travel. If the paper did not contain the phrase ‘treatment burden’ or ‘burden of treatment,’ then it was categorized into Group 3.
- Indirect measures (Group 3): No reference specifically to treatment burden in the text or ‘burden of treatment.’ However, there is some incorporated measure for one or two dimensions of treatment burden, as per the conceptual framework. Because treatment burden is still in the developmental stage of operationalization, we believed that the scoping review would have been incomplete without its inclusion. Although these papers do not specifically refer to the concept of treatment burden they measure one or two of the commonly accepted dimensions of the concept. Furthermore, this was in line with the aim of a scoping review, which aims to draw upon a diverse range of papers and provide a considerable summary of the relevant, available literature.

A summary of the data analysis framework used is provided in Fig. 2.

## Results

**Fig. 3:**
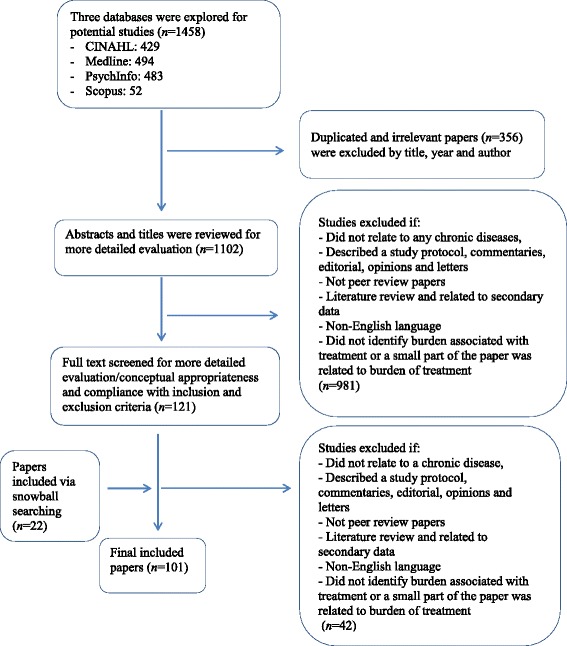
Overview of literature search and inclusion

Database searching identified 1458 potential papers. After removal of duplications, and irrelevant papers by title, 1102 abstracts remained. After applying the eligibility criteria, 121 papers remained for further investigation. An additional 22 papers were added via snowball searching. After screening was completed 101, full papers were included in the review. A flow chart describing the screening process is shown in Fig. 3. Included studies were published between the years 2000–2016; a quarter of the papers were published in 2015 (*n* = 26; 25.5%). The majority of the studies involved quantitative measures of treatment burden (*n* = 64; 63.4%). Twenty-eight studies (27.7%) were qualitative, and nine (8.9%) were mixed methods (see Table 1). One of these studies [18] involved mathematical modelling to identify how treatment burden influences treatment decisions.

**Table 1:** Study Characteristics

Characteristic		No. of studies
*Geographical Region*	Northern America	49
	Europe	18
	United Kingdom	16
	Asia-Pacific	10
	Other^a^	4
	Multi-site^b^	4
*Study Design*	Quantitative	64
	Qualitative	28
	Mixed Methods	9
	Tool Development or Validation^c^	20
*Sample size*	<50	28
	50–100	11
	100–500	32
	500–1000	9
	1000–10,000	15
	>10,000	6
*Study Participants* ^*d*^	Children & Adolescents	7
	Parents	6
	Adults (>18 years)	61
	Adults (>60 years)	12
	Children & Adults	10
*Groupings* ^e^	Group 1	16
	Group 2	50
	Group 3	35
*Therapeutic Area*	Cancer/Tumour	18
	Cardiovascular	5
	Diabetes	13
	Psychological	7
	Respiratory	17
	Dermatological	2
	Other^f^	15
	Co-morbidities or various	24
*Treatment Burden* ^g^	Medication	64
	Time/Travel	32
	Financial	22
	Healthcare Access	21
	Other: Lifestyle	16
	Other: treatment preferences/decision/	6
	Other: Interpersonal challenges	3
	Other: carer burden	3
*Measures* ^*h*^	Semi-structured questions	31
	Survey questions	24
	Prescription burden	15
	Disease specific tool^i^	28

^a^Israel, Turkey, South Africa and Jordan;

^b^Involving more than one country;

^c^Of all studies (counted twice)

^d^ *n* = 5 did not specify population, ‘children and adults’ accounted for studies involving children and their parents;

^e^Refer to Fig. 2 for more details about grouping

^f^Includes: chronic/end-stage kidney disease, liver transplant, spasmodic dysphonia, proliferative lupus nephritis, intensive care, gastroesophageal reflux disease, primary ciliary dyskinesia, coeliac disease, systemic lupus erythematosus, percutaneous endoscopic gastrostomy;

^g^Some papers included more than one dimension of treatment burden (will not add to 101). Other aspects of treatment burden were identified, e.g. dietary restrictions (lifestyle), the influence of treatment burden on treatment preferences (treatment preferences/decisions), responses from others (interpersonal challenges), and impact on carers (carer burden)

^h^Out of 98 papers – three studies did not use specific measures e.g. economic evaluation, videographic analysis, normalization process theory on previous interview data

^i^Included: versions of Cystic Fibrosis Questionnaire Revised (CFQ-R; *n* = 7); Childhood Illness Attitude Scales (CIAS), versions of Quality of Life Bronchiectasis (QOL-B) (*n* = 3 each); Burden of Insulin Treatment, Treatment Related Impact Measure-Diabetes (Trim-D), Retinopathy Treatment Satisfaction Questionnaire (RetTSQ), Survey of the Adolescent Treatment Experience (SATE), Willingness to Accept Life-Saving Treatment (WALT), Elderly Diabetes Burden Scale (*n* = 2 each); Patient Benefit Index - standard version for chronic skin diseases (PBI-S), Diabetes Medication Satisfaction (DiabMedSat), Functional Assessment of Chronic Illness Therapy-Treatment Satisfaction-Bone Treatment Convenience and Satisfaction Questionnaire (FACIT-TS-BTCSQ), Medication Cost Reduction Strategies (MCRS), Revised Illness Perception Questionnaire (IPQR), Treatment Burden Index (TBI), Side effect rating scale, Dermatology Life Quality Index (DLQI), GERD Treatment scale, Quality of Life in patients with primary ciliary dyskinesia (QOL–PCD), Insulin Treatment Experience Questionnaire (ITEQ), Markov model of diabetes outcomes (*n* = 1 each)

From the 101 studies, a large majority were from Northern America (*n* = 49; 48.5%). There were 34 papers (33.6%) from Europe (16, 15.8%, from the UK) and 10 papers (10%) from the Asia-Pacific. Four studies involved participants from more than one country [9, 19–21]. Other sites included: Israel, Turkey, South Africa and Jordan (please refer to Table 1).

The smallest sample size involved six adults in Baylor et al.’s [22] phenomenological study, with the largest involving a dataset of 1,424,378 people in Scotland [23]. Twenty-eight studies involved less than 50 participants, 23 of which used a qualitative study design. A total of six studies involved more than 10,000 participants. Five papers did not specifically state the age range of participants, while 61 papers 60.4%) focused on adults (18 to 60 years of age), 12 (11.9%) on adults over the age of 60, 7 (6.9%) on children and adolescents, 6 (5.9%) on parents and 10 (9.9%) on children and parents.

A range of chronic conditions were included, the majority of which were grouped into one of the following therapeutic areas: cancer, cardiovascular disease, diabetes, psychological, respiratory, dermatological, or other, for example, chronic kidney disease, coeliac disease, transplant, primary ciliary dyskinesia and intensive care unit. The majority of studies focused on people with multimorbidities (*n* = 24), followed by cancer (*n* = 18), respiratory conditions (*n* = 17), diabetes (*n* = 13), psychological (*n* = 7), cardiovascular (*n* = 5), dermatological (*n* = 2). Fifteen studies focused on other chronic health conditions. The majority of studies involving children (and/or parents) were for respiratory conditions, e.g. cystic fibrosis. In accordance with the data extraction strategy, papers were grouped into three categories and analyzed using this grouping system.

### Group 1 - direct measures

There were a total of sixteen papers in this group. All papers focused solely on understanding the burden of treatment among adults, with chronic health conditions, with the exception of Sav et al. [24], which explored the meaning of treatment burden among stakeholders and key non-government health organizations. Nine papers were qualitative based [2, 11, 21, 24–29], five were quantitative [6, 9, 30–32], and two were mixed-methods studies [33, 34].

Two of the qualitative based papers used Normalization Process Theory (NPT), which aims to explain how the work of engaging in some ensemble of activities is accomplished through the operation of four mechanisms: coherence, cognitive participation, collective action, and reflexive monitoring, to interpret and categorize their data analysis [2, 27]. Eton et al. [25] utilized this theory to inform interview questions in their study. Gallacher et al’s [2] paper was based on secondary data (originally undertaken to explore chronic heart failure), and the authors extracted information pertaining to treatment burden. Other methods of data analysis included content analysis, thematic analysis, framework analysis, and Karamanidou et al. [28] utilized Interpretive Phenomenological Analysis (IPA). A review of the qualitative papers indicates that the interview probe questions covered the following key dimensions of treatment burden in the literature: medication difficulties and/or experience, cost of treatment, relationships with health professionals, understanding of treatment and associated information needs, challenges/barriers to treatment and/or self-management, lifestyle restrictions, family relationships, treatment adherence and satisfaction. However, with the exception of Gallacher et al. [1], Karamanidou et al. [28], Tran et al’s [21] paper, and Eton et al’s [25, 26] papers, there was limited information about the interview process and the specific questions asked about treatment burden. Nevertheless, sample interview questions in some of these papers included: ‘where participants went for information regarding their illness,’ ‘how treatment restricted their daily activities,’ ‘what participants felt was the most difficult aspect to manage with their treatment and illness,’ ‘whether participants believed that it was important to follow their treatment regimen,’ and ‘whether participant’s felt that they knew enough about their treatments.’ Eton et al’s (2012) paper on building a measurement framework of treatment burden in complex patients with a range of chronic conditions was the most comprehensive in terms of detailing the questions covered in the interviews. The authors included an interview schedule in their paper, which consisted of 11 questions informed by previous studies of treatment impact/satisfaction [35] and NPT. The authors used the framework they developed in this study for their subsequent studies in 2015 and 2016, the latter being a quantitative validation of a 9 factor, 48-item treatment burden measure: the Patient Experience with Treatment and Self-management (PETS).

In contrast to the qualitative papers, those utilizing a quantitative design generally contained more descriptive information about how treatment burden was measured. For example, in 2006, Nordyke et al. [31] conducted a study to validate a Patient Satisfaction Questionnaire for Anemia Treatment (PSQ-An) in cancer patients. Although their study was primarily concerned with treatment satisfaction rather than burden, the latter was nevertheless measured via a 10-item scale. However, there was limited insight provided into how the treatment burden dimension of the PSQ-An was developed or the specific conceptual framework that was used. In another paper, focused on cancer patients, Henry et al. [32] investigated the side effects of chemotherapy or radiotherapy treatment; limited information was provided about how this was measured beyond referring to the 13-item Functional Assessment of Chronic Illness Therapy (FACIT) Fatigue Scale (version 4). Bohlen et al. [33] examined (by video-graphic data) whether people with type 2 diabetes and their clinicians discussed treatment burden, the characteristics of their discussions, and their attempts to address this burden during visits. Based on previous research, the authors identified four dimensions of treatment burden: access, administration, effects, and monitoring. Also in 2012, Tran et al. developed the Treatment Burden Questionnaire (TBQ) in France, which consisted of seven items (two of which had four sub-items) in the French language. Later, Tran et al. [21] adapted the TBQ amongst an English speaking sample using an internet platform. The TBQ in this paper assessed the following dimensions: (1) taking medication, (2) self-surveillance, laboratory tests, doctor visits, need for organization and administrative tasks, (3) following advice on diet and physical exercise, and (4) social impact of the treatment. The PETS measure, validated by Eton et al. [30] included the following dimensions: medication information, medications, medical appointments, monitoring health, interpersonal challenges, medical expenses, difficulty with healthcare services, role activity limitations, and physical/mental exhaustion.

### Group 2 - inferred measures

A total of 50 papers were identified in Group 2. Despite suggesting that they measured treatment burden, the papers in this group only included one or two established dimensions of treatment burden, e.g. financial costs, burden of time. The majority of Group 2 papers were quantitative studies (36 in total) [18, 19, 23, 36–68]. There were also nine qualitative papers [22, 69–76], and five mixed-method studies [20, 77–80].

There were wide variations within these papers with respect to their level of focus on treatment burden. Some papers suggested a focus on treatment burden by their study title, yet upon further inspection only included one or two commonly accepted dimensions of treatment burden such as medication or costs, for example, [68, 69]. Some authors calculated treatment burden with respect to the number of treatment episodes or in terms of level of treatment intensity [46, 51]. Others explored how treatment burden influenced treatment decisions [18, 70, 80] and developed an associated measure [80]. This measure, Willingness to Accept Life-Sustaining Treatment (WALT) was also utilized by Janssen et al. [44] for Dutch patients with two of the three conditions explored by Fried et al. [80]: chronic obstructive pulmonary disease and chronic heart failure. Other authors stated that they measured treatment burden but provided minimal or no detail on the actual measurement process or sub-scales used [20, 44, 48, 55, 56, 78]. Readers would therefore need to search beyond the paper to identify what dimensions of treatment burden were included. There were also some papers that referred to treatment burden but focused predominantly on experiences of living with a chronic condition, rather than treatment burden per se [73, 74], or alternatively, focused on both, i.e. the burden of symptoms and treatment, as seen in Liu et al. [47]. Finally, there were several papers that focused on understanding treatment experiences and included several broad or specific questions on treatment burden [20, 64]. Robertson et al. [63] was unique in that it surveyed regular medication users and the impact of financial and medication burden (in addition to other variables) on choosing to take a new medication.

The most common dimension of treatment burden examined in Group 2 was related to medication use, generally around diabetes management (e.g., blood glucose monitoring), e.g., [19, 38, 39, 49, 79], and other chronic diseases, such as asthma [71], attention deficit hyperactivity disorder [69] and stroke [23]. Based on the limited information on survey development in many studies, it appeared that medication burden was measured by asking participants questions related to their experiences of taking medication, including, but not limited to, side effects, inconvenience, e.g. from carrying, storing, and administering medication, and impact on lifestyle, e.g. diabetic medication and effect on meal times, exercise and/or diet [19, 48, 79]. Alternatively, one broad treatment burden question was included by Hanke et al. [43], who asked participants with actinic keratosis how much of a problem had the treatment been, ‘for example by making your home messy, or by taking up time?’ Some studies identified the total number of therapeutic drug classes or medications a person was taking, i.e. polypharmacy, as a sign of treatment burden [23, 49, 58–60]. Yet, there was acknowledgement by Gallacher et al. [23] that medication use represents only one dimension of treatment burden; other considerations include financial burden and healthcare access [23]. Five papers investigated the relationship between treatment burden (alongside a range of other variables) on medication adherence [50, 55, 61, 72, 75]. For example, a question asked to adolescents with cystic fibrosis was ‘are there other things, besides the time involved, that make it difficult to do your therapies or take your medicines?’ [72]. Similarly, Hock et al. [61] asked the following question: ‘Treatments have been burdensome on my family's resources, e.g. money, time, energies.’

Following medication, there was an emphasis on financial burden or the cost of treatment for participants and their families. For example, Colombo et al. [40] examined the medical costs (i.e. hospitalizations, outpatient medical interventions, medication, devices and dietetic products) associated with treating cystic fibrosis in Italy. Additionally, Kent et al. [45] investigated, via three questions, whether cancer survivors who experienced financial problems were likely to forgo or delay medical care, such as counselling, dental care, checkups, or prescription medication. Qualitative interviews with lung transplant recipients and health professionals identified treatment burden as a novel health-related quality of life dimension, including financial costs [76]. Among the papers that focused on financial burden, there was also attention on the cost of treatment upon the family unit, in addition to the patient [45]. Thus, it was evident that the cost of treatment, particularly medication, was a well-established concern for participants.

Within Group 2, there was significant interest in understanding the burden of treatment for cystic fibrosis, for children, adolescents, adults, and parents [36, 40, 52–54, 57, 65, 75]. A total of 12 Group 2 papers focused on cystic fibrosis, although the study by Ziaian et al. [68] also included participants with asthma and diabetes. The general focus of these studies was on quality of life; treatment burden was measured as part of living with cystic fibrosis, generally via two or three items. However, detailed information on this was missing from many papers. Most of these papers simply indicated that health related quality of life for cystic fibrosis was measured, which included physical functioning, treatment burden and respiratory symptoms. Nevertheless, the large number of papers that focused on cystic fibrosis confirmed that this particular chronic condition is associated with high treatment burden.

There was also a focus on investigating the burden of treatment on specific treatment interventions. Most of these papers measured treatment burden via one or two simple questions, which asked about general treatment experiences. These measures were not grounded in theory and lacked a clear focus on treatment burden. Rather, they were generally focused on the impact of a particular treatment intervention. For example, for two Randomized Control Trial (RCT) patient groups on varying medication for proliferative lupus nephritis, Grootscholt et al. [42] measured the associated burden of treatment via a 5-point Likert scale and used open-ended questions, for example, exploring which aspect of treatment was perceived as most burdensome. Baylor et al. [22] investigated treatment burden for spasmodic dysphonia by asking participants how Botox treatment affected their ability to do things they wanted to do. Levinson et al. [77] asked past ICU patients whether they thought their treatment was worthwhile. Finally, Drabe et al. [66] asked participants with thyroid cancer, and their partners, about how they experienced surgery and what they felt about the radioactive iodine treatment and associated treatment isolation.

### Group 3 - indirect measures

There were a total of 35 papers in this group; 23 of these were quantitative [81–103], 10 were qualitative [104–113], and two mixed-methods [114, 115].

Although Group 3 papers did not specifically mention treatment burden or burden of treatment, careful analysis suggested that they measured the dimensions of burden put forward by Sav et al’s [8] review, such as medication, time/travel and cost of treatment. Rather than concentrating on treatment burden, these papers generally focused on treatment experiences and included arbitrary questions that measured some dimensions of treatment burden. For example, Broom et al. [85] used a questionnaire with questions relating to bone treatment convenience, e.g. did treatment for bone disease take up family time and were participants concerned about associated side effects. Similarly, Passik [97] asked participants undergoing cancer treatment about common treatment side effects, such as weight loss, constipation, nausea, pain, or vomiting. DeSmedt et al. [88] asked heart failure patients about adverse drug events, without specifically discussing treatment burden. Eiser and Upton [89] aimed to provide estimates of the cost of caring for a child with cancer, without specifically discussing how the cost of treatment could be a critical dimension of treatment burden. In contrast to the afore-mentioned studies, Blome et al. [84] assessed the importance of treatment goals for people with moderate to severe psoriasis. Participants acknowledged the importance of low treatment burden with respect to time/travel and costs. A mixed-method study by Tijerina [115] explored the experiences of Mexican-American women receiving dialysis treatment, and identified a loss of identity from changes in their body image, loss of independence, freedom and ability to fulfil social roles. Furthermore, women who expressed concerns about body image were more likely to be non-adherent to treatment (Tijerina 2006).

As can be seen from the examples provided, a large number of studies (*n* = 22) focused on medication related factors, which is a commonly accepted dimension of treatment burden. Most of these studies aimed to understand the experiences of participants whilst using prescribed medication or treatments, such as dialysis or surgical procedures, and talked about issues, such as medication side effects, cost, inconvenience or changes in treatment, and feelings whilst taking medications. There were also instances where medication burden or polypharmacy was the general focus of the study and was calculated with the number of medications a person was taking, for example, using more than five medications was classified as burdensome [60, 92, 93], or by the number of medications dispensed or filled [82]. This, as noted by the authors, assumed that patients actually used what was supplied to them. In addition to medication, there was also a focus on cost of treatment [89, 102, 103] and time and travel burden [81, 86, 95, 96]. For example, United States (US) families at a higher risk of experiencing time burden from caring for children with chronic conditions included those from a lower income and educational background, were culturally diverse, lived in a rural location and had public health insurance [95]. Furthermore, the more severe the child’s chronic condition, the higher the risk of time burden. The issue of time and financial burden was also emphasised by caregivers of children with chronic conditions in a Canadian study [102]. Although these papers appeared similar to those in Group 2, the fundamental difference was that these papers did not specifically mention treatment burden in the text, and focused on investigating medication or treatment-related experiences. Alternatively, Group 2 papers mentioned the term ‘treatment burden’ and investigated medication related issues as a way of understanding the level of treatment burden.

Some of the studies focused on understanding the illness or treatment journey among participants with chronic conditions, particularly cancer [98, 108–111, 114]. For example, Shadid et al... [110] investigated the experiences and barriers of Aboriginal people in accessing cancer services and treatment. The authors found that transport, accommodation and travel expenses were a concerning issue for this specific population whilst undergoing cancer treatment. Similarly, Salter et al. [109] explored the perceptions of dialysis and kidney transplant among African American adults undergoing haemodialysis. Although Salter et al’s [109] paper did not specifically focus on treatment burden per se, the responses of participants during the focus group interviews clearly indicated episodes of treatment burden, with one participant admitting: *“I used to feel like Superman. I’m unhealthy. This dialysis thing is like kryptonite. It sucked everything out of me.”* Also, Clark et al. [114] explored treatment decisions by prostate cancer patients, which could be related to treatment burden, e.g. regretting treatment choice. Stajduhar et al. [111] wanted to understand the experiences and educational needs of Canadian adults receiving iodine therapy for thyroid cancer. In-depth responses to the question *‘tell me about your experiences when you were a patient on our unit,’* generated treatment burden-related responses, such as feeling socially and physically isolated during treatment. In a qualitative study, Bower et al. [104] interviewed 28 participants with various co-morbidities (including cancer) to identify how they perceived, and responded to, their condition/s, i.e. illness representations. The authors identified concerns related to medication burden, such as addiction to pain killers and quantity of medication prescribed.

Finally, in some papers, there was also a focus on reduction strategies that participants employed to minimize treatment burden. Burcu et al. [87] asked participants if they actively implemented strategies to reduce medication costs. For example had participants taken smaller doses, or skipped doses, of a medication to make it last longer, decided not to fill or refill a prescription because it was too expensive, or spent less money on food, heat or other basic needs to be able to afford medication, etc. Bower et al... [104] found that many patients took multiple medications for co-morbidity and when they prioritized a chronic condition, they also prioritized the medication used to treat that condition.

## Discussion

This scoping review reveals a significant variation in how the treatment burden experienced by those with chronic disease, has been operationalized and measured. These variations reflect the evolving and developing nature of the treatment burden literature, as well as the diversity of chronic conditions experienced. Indeed, research on treatment burden has significantly increased since May et al’s (2009) seminal work. This has led to the development of various measures to assess and understand the level of treatment burden experienced by those with chronic disease. However, our review indicates that despite this growing research interest and substantial progress, research gaps remain.

Firstly, our review highlights the dominance of quantitative based papers to understand and measure treatment burden; 64 of the 101 studies in this review were quantitative. Despite the widely known strengths of quantitative research, the limited use of qualitative methods limits our ability to understand, in-depth, treatment burden experiences among specific populations. For example, a qualitative approach may be preferred over a quantitative method, when there is a need to explore and understand, in-depth, how non-traditional and minority populations experience treatment burden. We believe that Eton et al’s [25] and Karamanidou et al’s [28] work provide a useful starting point for a focus on qualitative research. In their qualitative paper, Eton et al. [25] provided an 11 question interview schedule, which asks participants about their health condition/s and how they care for them, the impact of treatments and medical self-care on their daily life, difficulties accomplishing self-care, relationships with health professionals, financial problems and the factors that may alleviate treatment and self-care burden [26]. Additionally, Karamanidou et al’s [28] paper, which uses IPA, includes a valuable interview schedule, which can be easily amended to reflect the type of chronic condition under investigation. Researchers have much to gain from using exploratory qualitative research methods to further conceptualize treatment burden and understand how it occurs, before assessing levels of treatment burden among large populations. This is not to say that qualitative research should be conducted at the expense of quantitative research but rather, both should be utilized to obtain a more in-depth and comprehensive explanation. Therefore, further mixed-methods research would be highly valuable in this area. Additionally, the question remains as to whether all domains of treatment burden are equally important. While many studies included in this review focused on medication burden, further studies may be needed to identify if this is really the most important dimension of treatment burden, and whether this changes depending on study population.

Secondly, despite the plethora of quantitative based research, it is clear that creating a measurement questionnaire/survey to assess levels of treatment burden is still in its infancy phase. Although there has been increasing interest in treatment burden, our review indicates that only a handful of studies focus solely on the concept of treatment burden and offer systematic measures [6, 9, 21, 25, 26, 28, 30]. The primary focus for most Group 2 and 3 papers was on understanding the level of treatment burden as a component of overall treatment experience or the burden of living with a chronic condition. While this is valuable, most studies make little reference to treatment burden and simply measure it via one or two items or as an aggregated figure. However, previous research has shown [8] that treatment burden is a subjective concept and one person’s response to a particular treatment intervention may be very different to another person undergoing the same treatment. Research has suggested that individual capacity issues relating to the individual and their wider support networks will influence response and ability to cope with any given level of treatment burden [116]. Hence, it is likely to be inadequate to simply measure treatment burden by asking participants if they take more than five prescription medications, as seen in this review.

Additionally, it seems unlikely that asking participants to estimate their level of treatment burden via two or three questions will fully capture the multi-dimensional nature of treatment burden. At best, two or three questions on treatment burden would only be sufficient to aid understanding of one dimension of this concept, such as medication use. We recommend looking at the psychometric literature for guidance about the development and validation of questionnaires. This body of literature can offer guidance around the number, length, and wording of questions, data collection and pilot testing, validation and evaluation, reliability and validity. In the meantime, Eton et al’s [26, 30] recent PETS measure, along with Tran et al’s [9, 21] TBQ are promising and can be a valuable starting point in this area. However, we do acknowledge that trying to understand levels of treatment burden via lengthy surveys or long lists of questions may not always be possible because of time constraints, particularly in a clinical setting. Hence, it would be valuable to develop a short measure to identify levels of treatment burden in a clinical setting and implement strategies to alleviate its impact. This will not only be valuable in a clinical setting but also could accompany other measures focusing on high burden conditions, such as cystic fibrosis. A short measure of treatment burden could easily complement other measures where the focus is on understanding quality of life or the experiences of living with a particular chronic condition. However, we do caution against using short measures for scientific purposes, where the intent would be on understanding the dimensionality of treatment burden.

Thirdly, our scoping review highlights a lack of longitudinal designs to understand and measure treatment burden. Given that levels of treatment burden experienced by a person can change over time in response to disease severity and control and the development of other chronic conditions [8], the limited number of longitudinal studies could be viewed as problematic. Measuring treatment burden via cross-sectional study designs, as it has been done, impedes our ability to fully understand the dynamic nature of treatment burden.

Fourthly, our review has also confirmed the scant evidence of treatment burden in developing countries and culturally different populations. The overwhelming majority of studies have been conducted among Anglo-Saxon populations in affluent, high-income countries such as the United States, United Kingdom, Canada, Australia and France. Thus, there is limited knowledge about the experiences of people in different health contexts and among specific racial and cultural populations. The lack of research among such populations is problematic because understandings of key concepts, such as death, illness, health and treatment can be very different in non-traditional societies. For example, is it not uncommon for people in certain cultures in some countries (e.g. Ghana in Africa) to perceive illness as a form of retribution from their gods and not a condition which can be treated medically [117]. We caution researchers to be vigilant about the applicability of measures developed in Western societies to understand treatment burden in non-traditional societies and populations. Although this is important for testing the robustness of the measures and original conclusions in Western societies, it is unwise to assume that they can be uncritically applied elsewhere. Instead, what may be more suitable are qualitative exploratory research methods, such as in-depth interviews to conceptualize the meaning and measure levels of treatment burden for people from diverse cultural backgrounds.

Finally, we think that a fundamental theme that is missing from current measures of treatment burden is identity. Despite the recognition of the impact of treatment workload on everyday activities, how treatment burden influences a person’s identity is limited (Demain et al. [10] and Tijerina [115]). There is also scant evidence on whether there is gender, age or cultural differences in treatment burden. Identity, which can be highly associated with gender, may offer a missing link to explaining the subjective and dynamic nature of treatment burden. Sav et al’s [3] recent paper, suggested that younger adults with chronic condition/s experience greater levels of treatment burden, compared to older adults. Although the authors argue that there may be several explanations to this finding, one possible reason could be related to the issue of identity. More specifically, elderly adults may be more likely to accept treatment burden as a necessary evil brought on by old age and living with multimorbidity. This group may become accustomed to the treatment workload or have more time when they retire. In contrast, treatment burden may less socially sanctioned in younger adults, who are expected to foster a healthy, productive, and active identity. Considering identity in treatment burden may help unravel many of the nuances of this concept and further attention is warranted in this area. Importantly, this suggests future research needs to examine how the concept of treatment burden may differ depending on variables such as age, gender, or cultural setting.

To our knowledge, this is the most comprehensive review of the treatment burden literature to date. Yet, it has a several limitations that must be acknowledged. It is possible that we may have missed papers that measure this concept. For example, this review was restricted to English language only papers, and hence, may have missed key papers measuring treatment burden in non-English speaking samples. However, there is growing evidence that this is unlikely to be a major problem [118]. The paucity of studies reporting the issue of treatment burden in low income countries could be considered a further limitation. Quality appraisal of the papers was not undertaken, however this aligns with the intention of a scoping review [14] which aimed to be comprehensive in providing a narrative and descriptive account of the current state of evidence on measuring treatment burden.

## Conclusions

Although research on treatment burden is growing rapidly, there is still much ground to cover and work to be done. Outstanding research issues that need addressed include a greater qualitative focus, more research with non-traditional populations in developing countries, a greater emphasis on longitudinal studies and the consideration of the potential effects of “identity” on treatment burden.

## Abbreviations

(PSQ-An): Patient Satisfaction Questionnaire for Anemia Treatment
CINAHL: Cumulative Index to Nursing and Allied Health Literature;
FACIT: Functional Assessment of Chronic Illness Therapy Fatigue Scale
HIV/AIDS: Human immunodeficiency virus infection and acquired immune deficiency syndrome
IPA: Interpretive Phenomenological Analysis
NPT: Normalization Process Theory
PETS: Patient Experience with Treatment and Self-management
RCT: Randomized Control Trial
TBQ: Treatment Burden Questionnaire
UK: United Kingdom
US: United States
WALT: Willingness to Accept Life-Sustaining Treatment
